# Post-Transcriptional Regulation of Connexin43 in H-Ras-Transformed Cells

**DOI:** 10.1371/journal.pone.0058500

**Published:** 2013-03-11

**Authors:** Mustapha Kandouz, Jing Zhao, Andrew Bier, Sergio Di Marco, Irene Oviedo-Landaverde, Imed-Eddine Gallouzi, Gerald Batist

**Affiliations:** 1 Department of Pathology, Wayne State University School of Medicine, Detroit, Michigan, United States of America; 2 Karmanos Cancer Institute, Wayne State University, Detroit, Michigan, United States of America; 3 Montréal Centre for Experimental Therapeutics in Cancer, Segal Cancer Centre, Lady Davis Institute for Medical Research, Department of Oncology, McGill University, Montréal, Québec, Canada; 4 Rosalind and Morris Goodman Cancer Center, Department of Biochemistry, McGill University, Montréal, Québec, Canada; King Faisal Specialist Hospital & Research Center, Saudi Arabia

## Abstract

Connexin43 (Cx43) expression is lost in cancer cells and many studies have reported that Cx43 is a tumor suppressor gene. Paradoxically, in a cellular NIH3T3 model, we have previously shown that Ha-Ras-mediated oncogenic transformation results in increased Cx43 expression. Although the examination of transcriptional regulation revealed essential regulatory elements, it could not solve this paradox. Here we studied post-transcriptional regulation of Cx43 expression in cancer using the same model in search of novel gene regulatory elements. Upon Ras transformation, both Cx43 mRNA stability and translation efficiency were increased. We investigated the role of Cx43 mRNA 3′ and 5′Untranslated regions (UTRs) and found an opposing effect; a 5′UTR-driven positive regulation is observed in Ras-transformed cells (NIH-3T3^Ras^), while the 3′UTR is active only in normal NIH-3T3^Neo^ cells and completely silenced in NIH-3T3^Ras^ cells. Most importantly, we identified a previously unknown regulatory element within the 3′UTR, named S1516, which accounts for this 3′UTR-mediated regulation. We also examined the effect of other oncogenes and found that Ras- and Src-transformed cells show a different Cx43 UTRs post-transcriptional regulation than ErbB2-transformed cells, suggesting distinct regulatory pathways. Next, we detected different patterns of S1516 RNA-protein complexes in NIH-3T3^Neo^ compared to NIH-3T3^Ras^ cells. A proteomic approach identified most of the S1516-binding proteins as factors involved in post-transcriptional regulation. Building on our new findings, we propose a model to explain the discrepancy between the Cx43 expression in Ras-transformed NIH3T3 cells and the data in clinical specimens.

## Introduction

The importance of the Ras genes in cancer has been recognized for a long time [1]. Ras isoforms can transform cells and are often found to be mutated and constitutively activated in human tumors [2] even resulting in important therapeutic implications such as predicting response to chemotherapy [3]. At least 25% of all human tumors present Ras gene activating mutations [4]–[7]. For instance, mutational analysis shows that glycine to valine substitution at codon 12 in the Harvey form of Ras (H-Ras^val 12^) results in the loss of intrinsic GTPase function, constitutive activation of the protein, and its conversion into an oncogenic form [8], [9]. In other cancers, such as breast cancer, Ras signaling pathways can be activated, even in the absence of mutations. For instance, the ErbB2 receptor tyrosine kinase is activated in over 20% of human breast cancers. ErbB2 activation promotes oncogenic transformation, in part, by activation of Ras [10]–[12]. Also, although only less than 5% of breast cancers are associated with mutations in Ras, the activity of this oncogene is very important in the mammary tumorigenic process [13]. The oncogenic potential of Ras activation involves signaling through multiple transcription factors, as well as through regulation of post-transcriptional events including mRNA stability and translation efficiency. It has been demonstrated that the primary effect of Ras signaling on gene expression may occur mainly at the post-transcriptional rather than the transcriptional level [14]. Furthermore, among the mRNAs most affected are those encoding proteins involved in cell-cell interactions [14]. In the present work, we were interested in investigating the post-transcriptional regulation by Ras of Connexin43, a protein involved in cell-cell intercellular communication, which we have recently suggested as a potential therapeutic target [15].

The gap junction intercellular communications (GJIC) have a broad physiological function including the regulation of cell growth, cell differentiation, and the maintenance of tissue homeostasis [16]–[23]. They involve structures composed of proteins called connexins (Cx), through which a multitude of second messengers and small molecules are transported. The impairment of gap junctional intercellular communication (GJIC) is a common clinical marker of various diseases including cancer [23]–[26]. We and others have shown that Cx43 is undetectable in early stage human breast cancer tissue compared with adjacent normal tissue [26]–[29] as well as in ovarian cancer, lung cancer, and neuroblastomas [30]–[33]. Cx43 loss is believed to be among the earliest events by which transformed cells acquire independence from stimuli from neighboring cells. Some protooncogenes have been shown to alter regulation of GJIC and Cx43 [34]–[37]. The data for the effect of Ras are complex, because its signaling pathway is shared by a number of receptor kinases that have different effects on Cx43 expression [34], [38]–[42]. Contrary to the general belief that connexins expression is decreased in transformed cells, we found that Ras induces the expression of Cx43 in the NIH3T3 cells [43].

In a previous study, we examined the mechanisms by which the Ras signaling pathway regulates Cx43 gene at the transcriptional level and characterized the promoter sequence determinants of this regulation [43]. Transcriptional regulation, however, could not be invoked to explain the loss of Cx43 in clinical specimens. Here we provide data on Cx43 post-transcriptional regulation, as driven by the 3′ and 5′UTRs, in Ras-transformed cells, that could constitute a molecular model for the discrepancy between the NIH3T3 cellular system and the clinical specimens.

## Results

### H-Ras Regulates Connexin43 Expression at Post-transcriptional Levels

**Figure 1:**
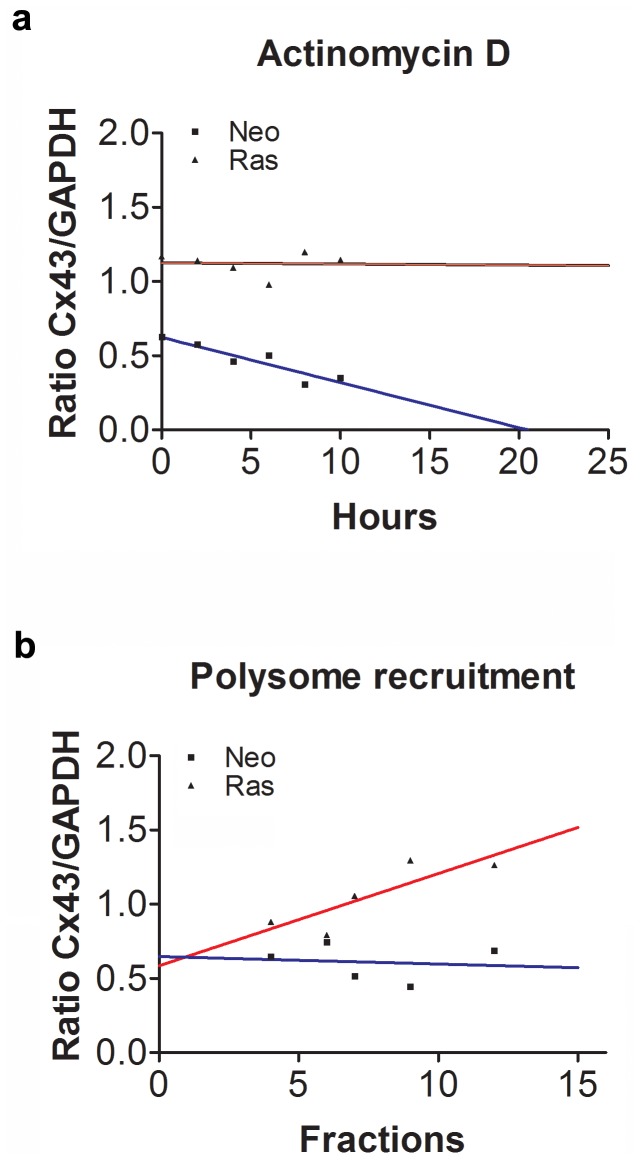
H-Ras regulates Connexin43 expression at post-transcriptional levels. ***a)*** RNA stability measured by the rate of Cx43 mRNA decay following inhibition of *de novo* transcription by actinomycin D. NIH-3T3^Neo^ and NIH-3T3^Ras^ cells were treated with actinomycin D (15 µg/ml), harvested at various time points and Cx43 and GAPDH mRNA levels were determined by RT−PCR. The values of Cx43/GAPDH mRNAs ratios are reported over the duration of treatment. The lines are based on a linear regression and indicate that there is a significant slope deviation from the zero for Neo (r = 0.84; *P*  = 0.0095) *versus* Ras (r  = 0.0012; *P = *0.94). ***b)*** Translation efficiency of the Cx43 mRNA. Polysomal and subpolysomal fractions were extracted from NIH-3T3^Neo^ (Neo) and NIH-3T3^Ras^ (Ras) cells using a sucrose density gradient and the relative levels of Cx43 and GAPDH mRNAs in these fractions were determined by RT-PCR. The results are reported as a Cx43/GAPDH mRNA ratio in different fractions. The lines are based on a linear regression and indicate that there is a significant slope deviation from the zero for Ras (r  = 0.014; *P*  = 0.84) *versus* Neo (r  = 0.71; *P*  = 0.07).

To study post-transcriptional regulation of Cx43 by H-Ras, we first examined RNA stability by measuring the rate of Cx43 mRNA decay following inhibition of *de novo* transcription by actinomycin D. NIH-3T3^Neo^ and NIH-3T3^Ras^ cells were treated with actinomycin D (15 µg/ml), harvested at various time points over a 12 h period and Cx43 mRNA levels were analyzed by RT−PCR. GAPDH mRNA levels were also analyzed by RT-PCR and used as a reference. The values of Cx43/GAPDH mRNAs ratios are reported over the duration of treatment. As shown in Figure 1a, the Cx43/GAPDH ratio decreases extremely faster in NIH-3T3^Neo^ cells (curve crossing the X axis at 20 hours) than in NIH-3T3^Ras^ cells (curve showing a plateau). Therefore, H-Ras overexpression induces a significant increase in Cx43 mRNA stability. We next addressed the effect of H-Ras on the translation efficiency of the Cx43 mRNA. Polysomal and subpolysomal fractions were extracted from NIH-3T3^Neo^ and NIH-3T3^Ras^ cells using a sucrose density gradient and the relative levels of Cx43 and GAPDH mRNAs in these fractions were determined by RT-PCR, as previously described by us [15]. The results are reported as a Cx43/GAPDH mRNA ratio in different fractions. Interestingly, we found that there is proportionately more Cx43 mRNA residing in the polysomal fractions (that are being translated) of the NIH-3T3^Ras^ cells than in the NIH-3T3^Neo^ cells (Figure 1b). Therefore, in addition to increasing Cx43 mRNA stability, H-Ras increases its translation efficiency.

### H-Ras Differentially Regulates Connexin43 mRNA 3′ and 5′Untranslated Regions

**Figure 2:**
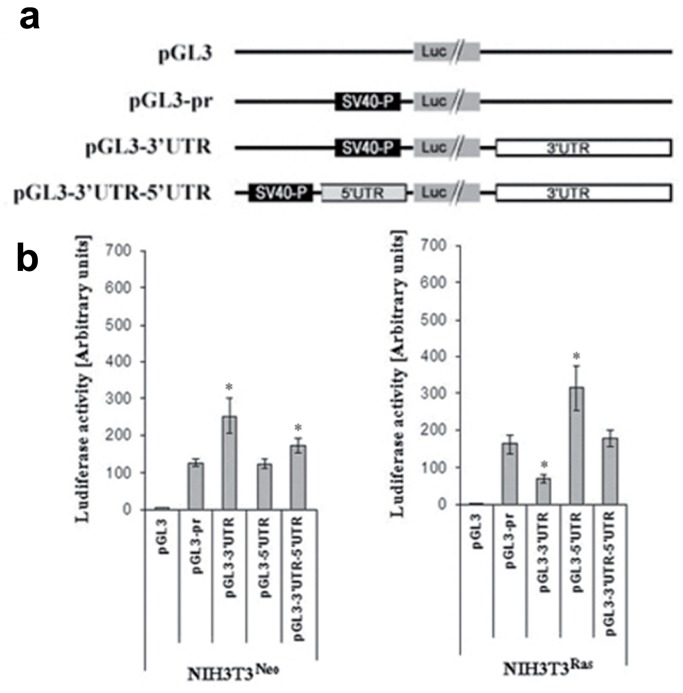
3′ and 5′UTRs-driven regulation in control *versus* Ras-overexpressing cells. ***a)*** Description of the different Cx43 mRNA 3′UTR and 5′UTR constructs used in this experiment. All constructs, except the pGL3 basic vector, contain the SV40 promoter (SV40-P) and the Luciferase coding region (pGL3-pr) in addition to the 3′UTR (pGL3-3′UTR) and/or the 5′UTR (pGL3-5′UTR) full length sequences. ***b)*** Luciferase assay using Cx43 mRNA 3′UTR and 5′UTR constructs in NIH3T3^Neo^ cells. The firefly luciferase activities were reported to the *Renilla* luciferase control values as explained in “Materials and Methods”. The experiments were performed at least three times in quadruplicates (*p<0.05).

Control elements involved in post-transcriptional regulation are mainly located within the mRNA 3′ and 5′untranslated regions (UTRs). In order to investigate post-transcriptional events involved in Ras-mediated regulation of Cx43, we first cloned the whole Cx43 3′UTR and 5′UTR from normal mammary epithelial cells by 5′- and 3′-Rapid Amplification of cDNA Ends (5′- and 3′-RACE). The entire 3′-UTR of Cx43 encompasses over 2200 bases (PubMed accession # NM_000165), which is over double of the average length of 3′UTRs in human mRNAs [44], [45], while the 5′UTR is much smaller (250 bp). We created reporter gene constructs by inserting the Cx43 3′-UTR and/or 5′UTR downstream or upstream of the luciferase gene coding region respectively (Figure 2a). In these 3′UTR and 5′UTR-driven reporter constructs, differences in luciferase activity reflect differences in regulation due to post-transcriptional events. After transfection of NIH3T3^neo^ and NIH3T3^Ras^ cells with these vectors, we found that while the 3′UTR induces the expression of the luciferase reporter gene in NIH3T3^neo^ cells, it significantly inhibits this expression in NIH3T3^Ras^ cells (Figure 2b). By contrast, the 5′UTR doesn’t affect the luciferase expression in NIH3T3^neo^ cells, while it significantly stimulates this expression in NIH3T3^Ras^ cells (Figure 2b). This result suggests that the 3′UTR and 5′UTR confer opposite regulatory functions to the Cx43 mRNA in the NIH3T3^neo^ *versus* NIH3T3^Ras^ cells. While the 3′UTR is stimulatory in the normal cells, the 5′UTR is stimulatory in Ras-transformed cells. Interestingly, it is the 3′UTR that shows a negative effect in transformed cells, an effect reminiscent of the observed loss of Cx43 expression in clinical specimens.

### The Cx43 mRNA 3′ and 5′UTRs Show Antagonistic Effects

We tested whether cooperativity or antagonism exist between the 3′UTR and the 5′UTR. A chimeric construct was made that contains both the 3′ and 5′UTRs (Figure 2a). Using this construct, we found that, although the 5′UTR doesn’t have an effect on its own in the NIH3T3^Neo^ cells, it moderately but significantly decreased the positive activity of the full length 3′UTR (Figure 2b). On the other hand, the full length 3′UTR dramatically counteracted the positive effect of the 5′UTR in the NIH3T3^Ras^ cells (Figure 2b). This result suggests that not only are the 3′UTR and 5′UTR differentially activated or inactivated in NIH3T3^Neo^ and NIH3T3^Ras^ cells, they exert an effect on each other and their effects are antagonistic.

### Ras Expression Exerts a Blockade on a Cx43 3′UTR-embedded Positive Regulatory Element

Based on the previous results, we hypothesized that the 3′UTR contains sequences that possess strong stimulatory effects in normal cells and that might be silenced in Ras-transformed cells.

**Figure 3:**
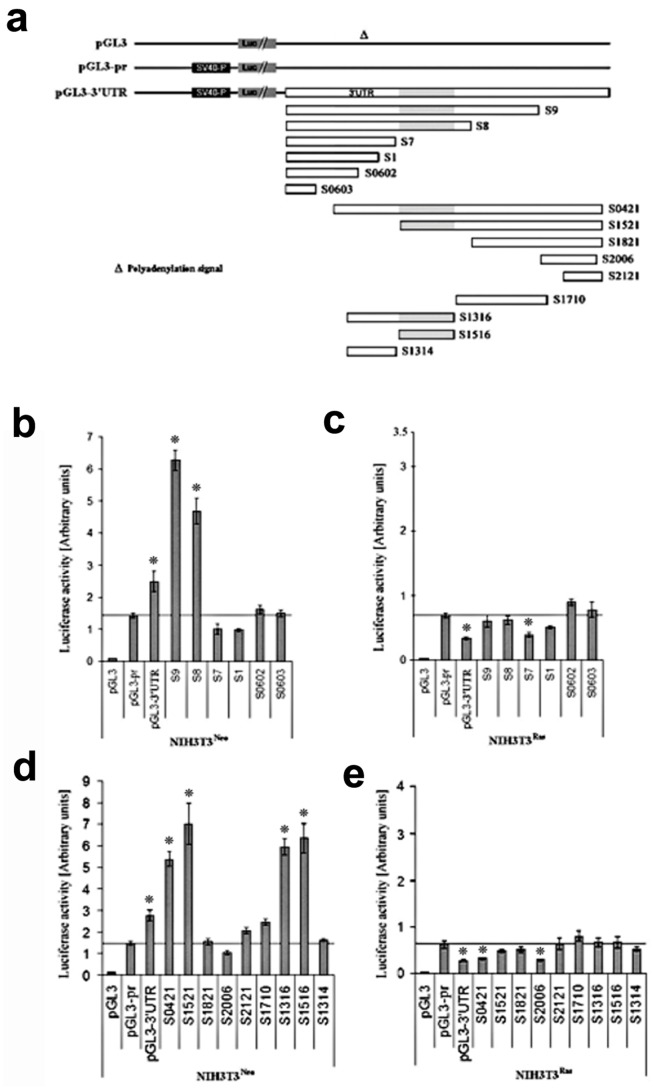
Characterization of the Cx43 mRNA 3′UTR regulatory regions in NIH3T3^Neo^ and NIH3T3^Ras^ by luciferase assay. ***a)*** Localization of the different Cx43 3′UTR constructs used in this work. All constructs contain the SV40 promoter (SV40-P) and the Luciferase coding region in addition to the pGL3 polyadenylation signal after the 3′UTR segments (not shown in graphics). The position of a putative polyadenylation signal is indicated. The S1516 region is shown in light grey. ***b, c)*** Luciferase assay of the first set of 3′UTR constructs used to transfect NIH3T3^Neo^ and NIH3T3^Ras^ cells respectively. ***d, e)*** Luciferase assay of the second set of 3′UTR constructs used to transfect NIH3T3^Neo^ and NIH3T3^Ras^ cells respectively. The firefly luciferase activities were reported to the *Renilla* luciferase control values as explained in “Materials and Methods”. The experiments were performed at least three times in quadruplicates (*p<0.05, reported to the pGL3-pr construct).

In order to determine these sequence determinants, we generated chimeric luciferase reporters with different segments of the Cx43 3′UTR. Using the full length 3′UTR sequence as a template, different regions were amplified and inserted downstream of the luciferase gene coding region. Each construct contained the luciferase coding sequence under the control of the SV40 promoter, followed by a Cx43 3′UTR portion (Figure 3a). In transfection assays using the NIH3T3^Neo^ cells, we found that serial deletions of the 3′UTR from the 3′ end (S9 and S8; Figure 3A) first resulted in an increase in luciferase activity that is much stronger than observed with the full length 3′UTR (Figure 3b), suggesting that the removed region contains a negative regulatory element. Further 3′ deletions (S7, S1, S0602, and S0603) resulted in an abrupt abolition of the positive effect and even a significant decrease in luciferase activity (S7, S1), suggesting that a strong positive regulatory element has been eliminated (Figure 3a,b). Strikingly, when transfected into NIH3T3^Ras^ cells all constructs resulted in a luciferase activity around or below the control pGL3-pr activity (Figure 3c). Based on the results of this first series of constructs, we made new constructs either deleted from the 5′ end or spanning the predicted positive regulatory region (Figure 3a). As shown in Figure 3d, 5′ end deletions (S0421, S1521) resulted in a strong increase in the luciferase activity in NIH3T3^Neo^ cells, more than observed with the full length 3′UTR, suggesting that a negative element has been deleted. Further 5′ deletions (S1821, S2006, and S2121) brought the luciferase activity back to the control level or even slightly lower (Figure 3d), confirming the presence of a strong positive element in this region. Fragments of the 3′UTR spanning the area of suspected positive regulatory activity (S1710, S1316, S1516, S1314), further localized this activity to S1516, a 337 nucleotides-long region in the median region of the 3′UTR (Figure 3a,d). Again, when transfected into NIH3T3^Ras^ cells, none of the sequences resulted in a significant increase in the luciferase activity, while some of them even decreased this activity similar to the effect the full length 3′UTR (Figure 3e).

These data demonstrate the presence of a strong stimulatory *cis*-acting regulatory element in the S1516 region of the 3′UTR of Cx43, whose activity is completely blocked in Ras-transformed cells.

**Figure 4:**
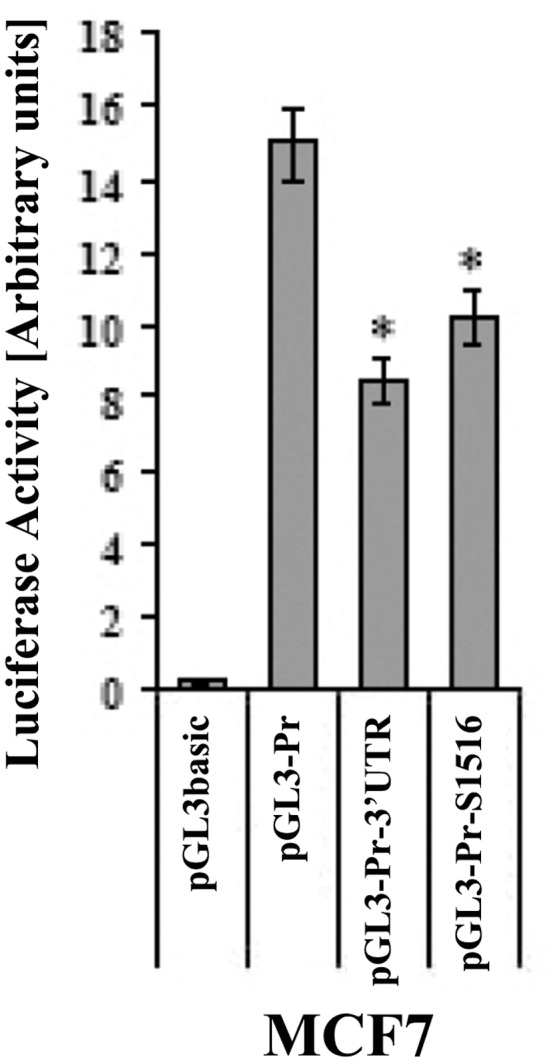
3′UTR and 5′UTR-driven effects in MCF7 breast cancer cells. Luciferase assay in MCF7 cells transfected with different constructs including the pGL3 control, the pGL3-Pr, in addition to the full length 3′untranslated region (pGL3-Pr-3′UTR) or the S1516 regulatory element (pGL3-Pr-S1516). The experiments were performed at least three times in quadruplicates (*p<0.05).

We next asked whether the observed 3′UTR and S1516 silencing effect could be found in a cancer cell line known for low expression of Cx43. Transfection of the breast cancer cell line MCF-7 resulted indeed in an inhibitory effect of both sequences (Figure 4), confirming that the 3′UTR S1516-mediated blockade of Cx43 expression is not tissue-specific but rather associated with the transformed state.

### Ras/Src and ErbB2 Activation Show Two Different Patterns of 3′UTR and 5′UTR-mediated Regulation

**Figure 5:**
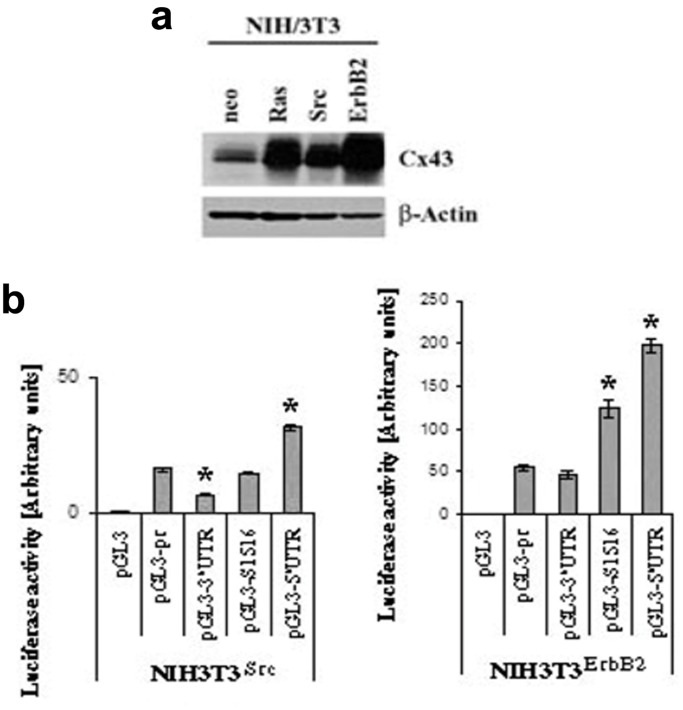
3′UTR and 5′UTR-driven effects in Src and ErbB2-transformed cells. *a)* Western blot of the Cx43 expression in protein extracts from NIH3T3^Neo^, NIH3T3^Ras^, NIH3T3^Src^ and NIH3T3^ErbB2^ cells. An antibody to β-Actin is used as a control for equal loading. ***b)*** Luciferase assay using the NIH3T3^Neo^, NIH3T3^Src^ and NIH3T3^ErbB2^ cells transfected with various Cx43 mRNA 3′ and 5′UTR constructs. The experiments were performed at least three times in quadruplicates (*p<0.05).

To investigate whether the effects observed in Ras-transformed cells are shared by other oncogenes, we analyzed Src and ErbB2-overexpressing NIH3T3 cells. Like NIH3T3^Ras^ cells, both NIH3T3^Src^ and NIH3T3^ErbB2^ cells were found to express high levels of Cx43 protein (Figure 5a). When NIH3T3^Src^ or NIH3T3^ErbB2^ cells were transfected with the full length 3′UTR and/or 5′UTR-bearing constructs, they behaved in a similar way as the Ras-overexpressing cells (Compare to Figure 2b): the 3′UTR (pGL3-3′UTR) lost its stimulatory effect, although to different extents (Figure 5b). Also, the 5′UTR (pGL3-5′UTR) had a strong positive effect in the Src or ErbB2-transformed cells (Figure 5b), not previously observed in the NIH3T3^neo^ control cells (Figure 2b). In particular, the 5′UTR-mediated effect is more pronounced in the NIH3T3^ErbB2^ cells than the other oncogene-transformed cells (Figure 5b). However, we observed an interesting difference: although the 3′UTR lost its positive effect in NIH3T3^ErbB2^ cells, it didn’t show any significant negative effect, unlike in the NIH3T3^Ras^ and NIH3T3^Src^ cells. In addition, the S1516 sequence showed a strong positive effect in both the control NIH3T3^Neo^ (Depicted previously in Figure 3) and the NIH3T3^ErbB2^ (Figure 5b) cells but not in NIH3T3^Ras^ (Depicted previously in Figure 3) and NIH3T3^Src^ (Figure 5b) cells. On the contrary, the S1516 sequence even showed a stimulatory effect in NIH3T3^ErbB2^ cells suggesting that in these cells, S1516 escapes the transformation-induced silencing. These data suggest the existence of at least two different Cx43 post-transcriptional regulation mechanisms involving the 3′ and 5′UTRs in Ras and Src-transformed cells on one hand and ErbB2-transformed cells on the other hand.

### Ras Activation is Associated with a Different Pattern of *trans*-acting Elements in the Cx43 3′UTR

**Figure 6:**
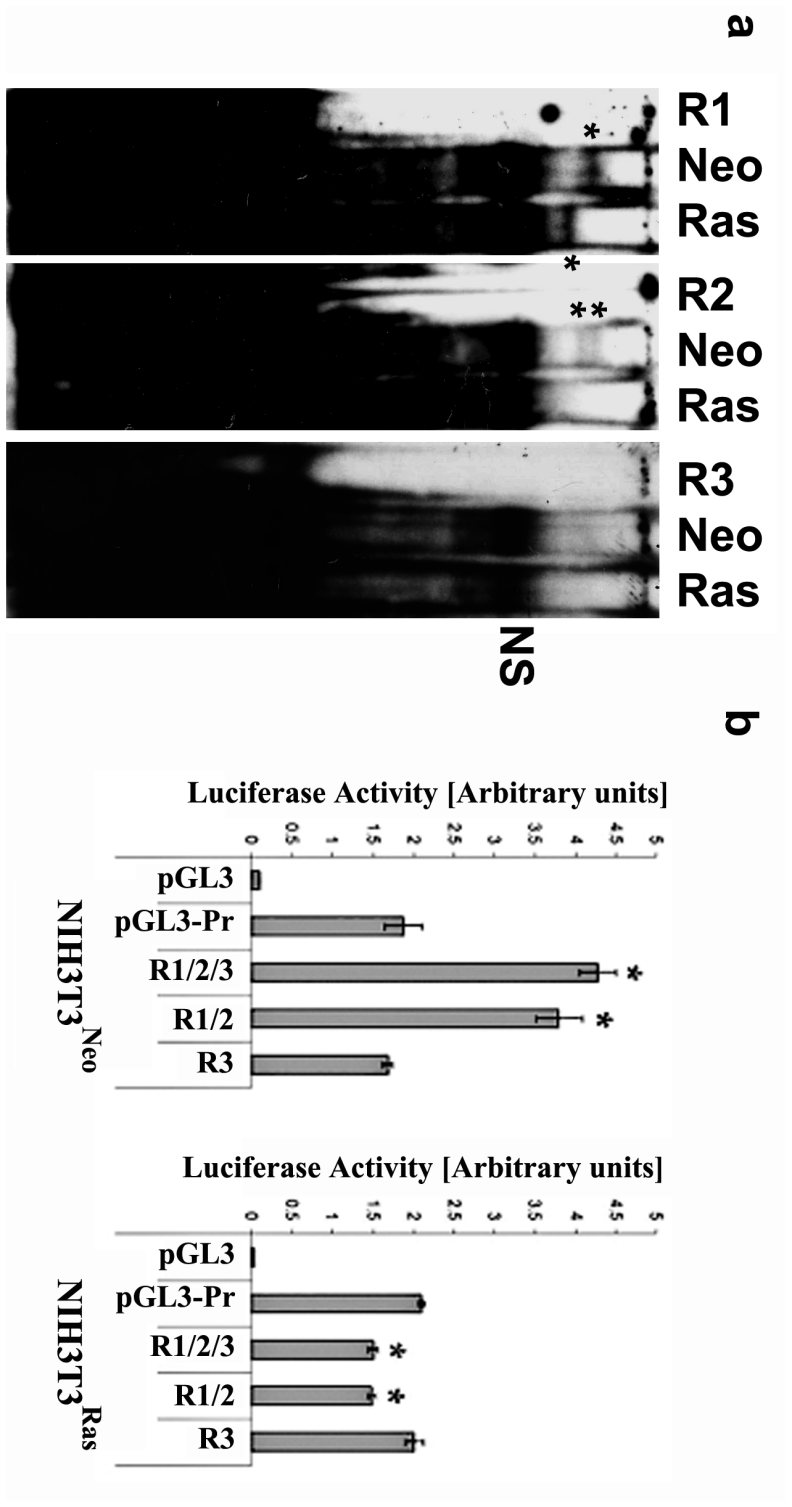
Differential binding of trans-acting factors to regions of the 3′UTR S1516. ***a)*** REMSA using three different riboprobes (R1, R2 and R3) spanning the S1516 region. Binding reactions were performed with the riboprobes as explained in “Materials & Methods” in the presence of protein extracts from NIH3T3^Neo^ (Neo) and NIH3T3^Ras^ (Ras) cells. RNA/proteins complexes are indicated by stars. N.S.: non-specific binding. ***b)*** Luciferase assay using the NIH3T3^Neo^ and NIH3T3^Ras^ cells, transfected with Cx43 mRNA 3′UTR constructs corresponding to the three riboprobes R1, R2 and R3 (*i.e.* full length S1516 element, R1/2/3), riboprobes 1 and 2 (R1/2) or riboprobe 3 (R3). The experiments were performed at least three times in quadruplicates (*p<0.05). The experiments were performed at least three times in quadruplicates (*p<0.05).

To determine whether the effects observed in *cis*-acting 3′UTR elements are associated with differences in *trans*-acting mRNA-binding proteins in either NIH3T3^neo^ or NIH3T3^Ras^ cells, we performed RNA electromobility shift assays (REMSA) to detect mRNA-protein complexes. We used 3 riboprobes corresponding to three overlapping regions (R1, R2 and R3) of the 337 nucleotides-long S1516 sequence in the presence of protein extracts from NIH3T3^Neo^ and NIH3T3^Ras^ cells. We observed the formation of different patterns of RNA-protein complexes in both cell lines (Figure 6a). Using the R1 riboprobe, both cells showed a single complex but whose size is larger (slower migrating) in NIH3T3^Neo^ cells. The R2 riboprobe gave two distinct complexes only in NIH3T3^Neo^ cells. The R3 Riboprobe didn’t show any differential binding. A strong band was found in all instances and was therefore labeled as nonspecific to any of the cell lines or any of the probes. These results clearly indicate the occurrence of different Cx43 mRNA 3′UTR-protein interactions in control *versus* Ras-transformed cells. To further confirm the confinement of the regulatory elements to regions R1 and R2, but not R3, we made two reporter constructs encompassing regions R1 and R2 on one hand and region R3 on the other hand. When tested by luciferase assay, and in accordance with the REMSA results, the R1/2 construct showed a strong increase in activity in NIH3T3^Neo^ cells, while it decreased the activity in NIH3T3^Ras^ cells (Figure 6b). The R3 construct didn’t show any change in the activity, just like the corresponding R3 riboprobe didn’t show any differential binding in REMSA experiments. Therefore we further restricted the S1516-born stimulatory activity to the R1 and R2 sub-regions.

### Ras Activation Results in Recruitement of Different hnRNPs and Intermediate Filaments to the Cx43 3′UTR S1516

To determine what *trans*-acting factor(s) bind the Cx43 3′UTR S1516 region, we synthesized biotinylated RNA probes corresponding to sub-regions R1 and R2. These probes were used to fish RNA-binding proteins (RBPs) using the magnetic field-based streptavidin/µMACS system, from NIH3T3^Neo^ and NIH3T3^Ras^ cells. We performed an SDS-PAGE and Coomassie blue staining of the isolated RBPs and we excised out the most variably expressed protein spots, which were next identified by mass spectrometry (LCMS/MS). We found that the list of R1-R2-binding proteins includes two main categories of proteins (Table 1): intermediate filaments (Lamins and Vimentin) and Heterogeneous nuclear ribonuclearproteins (hnRNPs K and M), in addition to the RNA helicase DDX17 and the chaperone heat shock cognate 71 kDa protein. Among the identified proteins are also two isoforms of a poly(C) binding protein (PCBP, also known as αCP or hnRNPE), PCBP1 and PCBP2, as well as an RNA helicase DDX17, a chaperone HSC70 and a paraspeckle protein 1.

**Table 1:** Major proteins that differentially bind the S1516 element of the Cx43 3′UTR in NIH3T3^neo^ *versus* NIH3T3^Ras^ cells, as identified by LCMS-MS.

Protein name	Function	Unigene ID
Lamin A/C	Intermediate filaments	Hs.594444
Isoform A of Lamin-A/C	Intermediate filaments	Hs.594444
Isoform C of Lamin-A/C	Intermediate filaments	Hs.594444
LMNB1 protein	Intermediate filaments	Hs.89497
Vimentin	Intermediate filaments	Hs.642813
paraspeckle protein 1 isoform alpha	RNA binding protein	Hs.213198
heterogeneous nuclear ribonucleoprotein M isoform a	hnRNP	Hs.465808
Isoform 1 of Heterogeneous nuclear ribonucleoprotein K	hnRNP	Hs.695973
Poly(rC)-binding protein 1 [PCBP1]	hnRNP	Hs.2853
Poly(rC) binding protein 2 [PCBP2]	hnRNP	Hs.546271
Isoform 1 of Probable ATP-dependent RNA helicase DDX17	RNA helicase	Hs.528305
Isoform 1 of Heat shock cognate 71 kDa protein	Chaperone	Hs.180414

## Discussion

**Figure 7:**
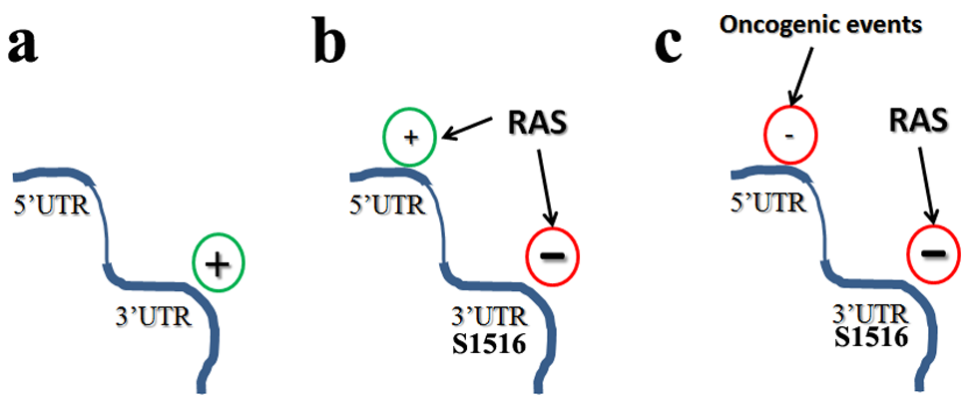
Model of the post-transcriptional regulation of Cx43 expression in normal and transformed cells. ***a)*** In normal cells, the 3′UTR exerts a positive regulation on Cx43 expression. ***b)*** Ras-mediated transformation exerts a blockade on a regulatory element (S1516) embedded within the 3′UTR, but releases a positive regulation by the 5′UTR, with a final result of induction of Cx43 expression. ***c)*** In human cancer cells, additional oncogenic events inhibit the 5′UTR-mediated regulation, which combined with the S1516-mediated blockade, results in the silencing of Cx43 expression in cancer cells.

In this work, we have studied post-transcriptional regulation of Cx43 gene expression in Ras-transformed cells. Using the cellular model of Ras-transformed NIH3T3 cells allowed us to unmask important determinants of the Cx43 post-transcriptional regulation. In this model, it was always surprising to us to see that Cx43 expression is increased in transformed cells, while the general belief is that Connexins are lost in cancer, as we observed in breast clinical specimens [26]. Therefore we underwent this study to identify gene regulatory elements that could explain this paradox. In summary, our results support a model (Figure 7) whereby: 1) The 3′UTR is responsible for Cx43 loss of expression in transformed cells, 2) We identified a strong regulatory element S1516which could account for this 3′UTR effect, since it presents a strong regulatory element that is positive in the normal NIH3T3^neo^ cells while it is negative in transformed NIH3T3^Ras^ cells. In other words, it is as if, in normal cells the positive regulatory function of S1516 results in increased Cx43 expression. However, following Ras-mediated transformation, the S1516 function is subject to a strong blockade and thus the loss of Cx43 expression, 3) Cx43 gene regulation shifts from a 3′UTR-driven positive regulation to a 5′UTR-driven positive regulation. This occurs after the strong S1516 positive regulation element is blocked in Ras-transformed cells, in parallel with the release of a 5′UTR-mediated positive function. The significance of shutting down the 3′UTR in the Ras-transformed cells is not clear, but this mechanism could explain the loss of Cx43 expression in clinical specimens. While this model is tempting, there remains an apparent paradox. At first view, the data regarding the 3′UTR silencing in NIH3T3^Ras^ cells suggests that the 5′UTR positive regulation, not the 3′UTR, might contribute to the increase in Cx43 expression in NIH3T3^Ras^ cells, in addition to the contribution of transcriptional elements that we previously identified [43]. Nevertheless, the hypothesis that the major event might be the silencing of a positive element or reactivation of a negative element is more consistent with the clinical observations: unlike the promoter region or the 5′UTR of the Cx43 mRNA, the 3′UTR is the only determinant that shows positive regulation in normal NIH3T3 cells that is turned off after transformation. For this reason, we focused on the 3′UTR-driven regulation, with the working hypothesis that, due to multiple transformation events (oncogene activation, tumour suppressor loss) the 5′UTR positive regulation, but most importantly the 3′UTR-driven positive function are silenced, therefore decreasing Cx43 expression (Figure 7). The fact that the cellular model of Ras-transformed NIH3T3 shows a silencing of S1516 but still shows higher levels of Cx43 might be due to the absence of additional oncogenic events which would silence the 5′UTR positive regulation, while Ras is responsible for silencing the S1516 regulatory element.

While the importance of the 5′UTR in regulation of Cx43 expression has previously been studied in three major papers [46] [47], [48], ours is the first study of the role of 3′UTR in Cx43 regulation in cancer. It is noteworthy that, as illustrated by our deletion study of the 3′UTR (Figure 3) showing significant activities in sequences other than the S1516 region, there are very likely other regulatory elements that await investigation. In the present study we focused on the element with the strongest activity. It is notable that the Cx43 mRNA presents a putative polyadenylation signal upstream of the S1516 region (Figure 3A). One way for the cells to regulate their Cx43 expression post-transcriptionally would be to alternatively use this poly(A) signal, thus generating a transcript devoid of the S1516-embedded regulatory element. Of particular interest, we have shown that the Cx43 pseudogene is expressed and functional in cancer cells [23], [49] and that it acts as a translational regulator of Cx43 expression [15], [23]. We are investigating the possibility that this regulation involves any of the 5′UTR or 3′UTR *cis*-acting elements elucidated in the present work. Although speculative at this point, we are also examining the hypothesis that the S1516 regulatory element might be targeted by a microRNA-mediated silencing. There is increasing evidence that Cx43 post-transcriptional regulation, specifically at the translational level, could involve specific microRNAs [50]–[53]. Proteins involved in Cx43 gene regulation might act through modulating this process. For instance, Dead end-1 (Dnd1), an RNA-binding protein, was shown to interfere with binding of microRNAs miR-1 and miR-206 with the 3′UTR of Cx43 [54]. If the Ras/S1516/microRNA hypothesis proves true, it would be possible to use microRNAs or their antagonists, to modulate the regulation of Cx43 expression by H-Ras and other oncogenes. This possibility would have an impact on the way we consider the therapeutic relevance of Cx43 and gap junction intercellular communication (GJIC) in cancer. Since GJIC in turn regulates microRNA transfer between cells [55]–[57], it would be conceivable to use this property to optimize the Cx43 targeting.

Among the proteins we identified in the ribonucleoprotein complex associated with the Cx43 mRNA 3′ UTR S1516 stimulatory element are many heterogeneous nuclear ribonucleoproteins (hnRNPs), including two isoforms of a poly(C) binding protein PCBP1 and PCBP2, hnRNPK and hnRNPM. The role of these proteins in post-transcriptional regulation (mRNA stabilization, translational activation or silencing) is well established [58]–[60]. Intriguingly, among these proteins we also found Lamins as major components of the 3′UTR-binding proteins which, to the best of our knowledge, constitutes a novel finding. There are reports of Lamins being involved in the spatial organization of RNA splicing factors in the nucleus [61], [62] and in modulating transcription factors functions [63]. Although speculative at this point, it is possible that interactions with lamin A/C and the nuclear matrix may be important for post-transcriptional regulation and the function of RNA-binding proteins. Similarly, Vimentin, another intermediate filament that we identified, has been previously shown in a single report to possess a function in gene regulation, by binding and stabilizing collagen mRNA [64]. It has also been identified in the nucleolus [65], a nuclear sub-compartment mainly known as pre-ribosomal RNA (rRNAs) splicing regulator but also reported to host processing and assembly of other non rRNAs [66]. Alternatively, it has become evident that the splicing process plays an important role in regulating RNA stability and translation [67]–[69] and it is therefore possible that a function of Vimentin could involve a splicing-based mechanism. Another of our mass spectrometry-identified proteins, DDX17, a member of the helicases, a group of multifunctional proteins implicated in unwinding of duplex RNA, has been described as a transcriptional co-activator or repressor [70], [71] and also as involved in pre-mRNA splicing [72], [73]. Within the S1516-binding proteins, we also identified the heat shock cognate 71 kDa protein (also called HSC70 or HSP70) was also assigned a chaperone role in RNA decay regulation [74]. Interestingly, it has been shown that Ras regulates the binding potentials of HSC70 to Bim mRNA 3′UTR, resulting in the latter’s destabilization [75]. Finally, very little is known about the paraspeckle protein 1 isoform alpha, except that paraspeckles have been described as ribonucleoproteic nuclear compartments with potential role in gene expression regulation [76], [77]. Taken together, our proteomics results suggest that the Cx43 3′UTR S1516 function involves binding to a battery of multifunctional RNA-binding proteins (RBPs). Identification of the RBPs which are differentially complexed with the Cx43 mRNA S1516 strong stimulatory element in normal *versus* Ras-transformed cells represents a significant step in understanding posttranscriptional regulation of Cx43 gene expression during the tumorigenic process. While the principal scope of this study was to characterize the structural *cis*-elements involved in post-transcriptional regulation of Cx43 expression during Ras transformation, a detailed mechanistic analysis of the role of the *trans*-acting factors identified by mass spectrometry promises to be a challenging and exciting avenue.

## Materials and Methods

### Cell Lines and Reagents

The mouse fibroblast stable cells NIH3T3^Neo^ (mock-transfected), the NIH3T3^Ras^, NIH3T3^Src^, and NIH3T3^ErbB2^ (stably expressing the constitutively active oncogene H-Ras-V12, Src and ErbB2 respectively) were obtained from Dr. Stephane Richard (Lady Davis Institute, Montreal, Canada) and were grown in Dulbecco’s modified Eagle’s medium with 10% calf serum and 1% penicillin/streptomycin at 37°C in a 5% (v/v) CO_2_ atmosphere. The luciferase vectors pGL3 (basic promoterless vector containing firefly luciferase), pGL3-pr (bearing an SV40 promoter) and pRL-Null (promoterless vector containing *Renilla reniformis* luciferase) were purchased from Promega (Madison, WI). The rabbit anti Cx43 antibody, recognizing nonphosphorylated and phosphorylated forms, was purchased from Zymed Laboratories (South San Francisco, CA). The anti- β-Actin and anti-Ha-Ras antibodies were obtained from Santa Cruz Biotechnology (Santa Cruz, CA). The anti-phospho-ErbB2 antibody was purchased from Upstate.

### Reporter Constructs and 5′- and 3′-Rapid Amplification of cDNA Ends (5′- and 3′-RACE)

The 5′/3′RACE kit (Roche) was used for the cloning of Cx43 mRNA 3′ and 5′UTRs following the manufacturer’s instructions. cDNAs were made using mRNA from normal human mammary epithelial cells (Clonetics) and used as templates in nested PCRs using adapter sequence-specific primers (included in the kit) and gene-specific primers. The pGL3-pr construct (pGL3 basic with an SV40 promoter, Promega) was modified to insert a *Pst*I restriction site, next to the *Xba*I site, between the Luciferase open reading frame (ORF) and the SV40 PolyA sequence. The resulting construct is called pGL3-prXP. We created reporter gene constructs by inserting DNA fragments encoding the 3′-UTR of human Cx43 mRNA into the *Xba*I/*Pst*I sites of pGL3-prXP. Portions of the 3′UTR were amplified by PCR and also cloned into the *Xba*I/*Pst*I sites of pGL3-prXP. The 5′UTR sequence was inserted between the SV40 promoter and the luciferase open reading frame. The sequences of the primers used in this study are available upon request.

### Polyribosome Analysis

Cells were treated with Cycloheximide (100 µg/ml) for 15 minutes at 37°C, washed 3 times with PBS containing cycloheximide (100 µg/ml), scraped and lyzed in lysis buffer (150 mM NaCl, 10 mM Tris-HCl pH 7.4, 0.5% Nonidet P-40, 10 mM MgCl_2_, 100 µg/ml cycloheximide, 2 mM DTT, 100 U/ml RNA Guard). Polysome purity and analysis was performed as previously described by us [15]. Briefly, lysates were spun down at maximum speed for 10 minutes at 4°C and supernatants are loaded onto a Sucrose Gradient (Light Sucrose (10%) and Heavy Sucrose (45%) solutions). After a 36K centrifugation for 2 hours at 4°C in SW40 rotor, fractions were collected using a fraction collector and stored on dry ice. The RNA from each fraction was extracted using a Phenol Chloroform extraction protocol and analyzed by RT-PCR for Cx43 and GAPDH using the following primers in Table 2.

**Table 2:** List and sequences of the oligonucleotides used in this study.

Name	Sequence
Cx43- for	5′-ATGAGCAGTCTGCCTTTCGT-3′
Cx43-rev	5′-AAGGGTCGCTCTTTCCCTTA-3′
GAPDH-for	5′-CCGGGAAACTGTGGCGTGAT-3′
GAPDH-rev	5′-GAAGGCCATGCCAGTGAGCT-3′
R1-for	5′-AATTCAGACAAGGCCCACAG-3′
R1-rev	5′-GTAGTAGCTGAGGAATGATG-3′
R2-for	5′-CATCATTCCTCAGCTACTAC-3′
R2-rev	5′-ATTAAGCATGGCTTGATTCC-3′
R3-for	5′-GGAATCAAGCCATGCTTAAT-3′
R3-rev	5′-CACCATATGTGCATTATTTT-3′
T7 promoter	5′-GAATTGTAATACGACTCACTATAGGGCGA-3′
R1Bio-for	5′-AATTCAGACGCGGCCGCCAGAA-3′
R1Bio-rev	5′- GTGAGTACTAGTTGAGGAATGATG-3′
R2Bio-for	5′- CATCATTCCGCGGCCGCTACTC-3′
R2Bio-rev	5′-ATTAAGCATGACTAGTTTCCCTG-3′
Bio-oligo	CATGGCGGCCGGGAGCATGCGACGTCGGGCCCAATCGCCC-bio

### Luciferase Assays

Cells were seeded in 24-well plates at a density of 40,000 cells/well and were incubated overnight. The following day, 1 µg of DNA was cotransfected with 0.2 µg pRL-Null and 4 µg of LipofectAMINE (Invitrogen) in 200 µl of serum-free media per well. After 5 h, the transfection mix was removed and cells were overlaid with 400 µl of complete medium per well. Cells were allowed to recover overnight and lysed 24 h after transfection. Lysis was performed and the dual luciferase activity was assessed by the dual-luciferase reporter assay (Promega) using the Lumat LB-9507 luminometer (PerkinElmer Instruments, Rodgau-Juegesheim, Germany). Luciferase activity was calculated as the ratio of firefly luciferase activity (of the promoter luciferase construct) to *R. reniformis* luciferase activity (of the vector pRL-Null). All assays were done at least in quadruplicate, and all transfections and luciferase assays were repeated in at least three independent experiments.

### RNA Elecromobility Shift Assays

The Cx43 cRNAs were generated from synthetic oligonucleotides (Region 1: R1-for and R1-rev; Region 2 : R2-for and R2-rev; Region 3 : R3-for and R3-rev) fused to a T7 promoter (T7p) by an *in vitro* SP6/T7 transcription kit (Roche) according to the manufacturer’s instruction (Sequences in Table 2). Briefly, the transcription reaction containing 1 µg of template, 2.5 mM ATP, GTP, and CTP, 200 µM UTP, 5 µl [α-^32^P]UTP, and 10 units of T7 RNA polymerase was incubated for 30 min at 37°C and treated with DNase I to get rid of the template DNA. The radiolabeled probes were purified using spin columns. The radiolabeled riboprobes were incubated with total extracts from NIH3T3^Neo^ and NIH3T3^Ras^ cells, in buffer containing 20 mM HEPES pH 7.6, 75 mM NaCl, 1.5 mM KCl, 5 mM MgCl_2_, 175 mM sucrose, 2 mM DTT and protease inhibitors (1 mM PMSF, 4 µg/ml of each aprotinin and leupeptin) at room temperature for 15 min. RNase T1 and heparin sulphate were added to final concentrations of 3000 U/ml and 5 mg/ml, respectively, and the mixture was incubated for an additional 15 min. Gel-shift/supershift assays were then performed by resolving the samples by SDS–PAGE.

### Isolation of RNA-binding Proteins

NIH3T3^Neo^ and NIH3T3^Ras^ cells (30–40×10^6^) were collected and suspended in 1 mL EBMK pH7.6 buffer [Hepes pH7.6 25 mM, MgCl2 5 mM, KCl 1.5 mM, NaCl 75 mM, Sucrose 175 mM] containing 0.5% NP-40, Complete protease inhibitor without EDTA (Roche) and DTT 2 mM. After sonication 3 times for 10 s at 200 W, the samples were centrifuged 15 min at 12000 rpm. For the preparation of biotinylated probes, sequences corresponding to the R1 and R2 regions of the Cx43 3′UTR were PCR-amplified using the primer sets: R1Bio-for and R1Bio-rev primers for R1 and: R2Bio-for and R2Bio-rev for R2 (Table 2) and cloned in the Not and SpeI sites of the pGEM-T Easy vector (Promega). A biotinylated oligonucleotide (Bio-oligo, Table 2) and 5–10 µg of pGEM-T-Easy–R1 or pGEM-T-Easy–R2 DNA template were used to synthesize RNA probes using the Ribomax large scale RNA production system T7 (Promega) as directed by the manufacturer. Next, RNA binding proteins were isolated from the total protein extracts in the EBMK buffer, using a protocol based on the use of magnetic beads attached to streptavidin and that specifically bind to the biotinylated RNA probe/RNA-binding proteins complex (µMACS streptavidin kit, Miltenyibiotec, Germany) following the manufacturer’s instructions. The R1 or R2-binding proteins were eluted from the columns using the elution buffer suggested by the manufacturer (Miltenyibiotec, Germany) and submitted to SDS-PAGE electrophoresis. The gels were stained with coomassie blue and the differentially present protein bands were cut out and used for mass spectroscopy identification. LCMS-MS was serviced at the IRIC (Institute for Research in Immunology and Cancer, Montreal, Qc, Canada) Proteomics and Bioanalytical Mass Spectrometry facility.

### Statistical Analysis

Statistics were conducted using the Student ***t*** test. ***P*** values less than 0.05 (*P<0.05) were considered to be statistically significant. Results are displayed as averages with error bars indicating standard deviations (SD). When indicated, statistics were conducted using GraphPad Prism 5.0 for Windows (GraphPad Software) to determine the linear regression and *P* values.
